# Prion neuropathology follows the accumulation of alternate prion protein isoforms after infective titre has peaked

**DOI:** 10.1038/ncomms5347

**Published:** 2014-07-09

**Authors:** Malin K. Sandberg, Huda Al-Doujaily, Bernadette Sharps, Michael Wiggins De Oliveira, Christian Schmidt, Angela Richard-Londt, Sarah Lyall, Jacqueline M. Linehan, Sebastian Brandner, Jonathan D. F. Wadsworth, Anthony R. Clarke, John Collinge

**Affiliations:** 1 MRC Prion Unit and Department of Neurodegenerative Disease, UCL Institute of Neurology, Queen Square, London WC1N 3BG, UK

## Abstract

Prions are lethal infectious agents thought to consist of multi-chain forms (PrP^Sc^) of misfolded cellular prion protein (PrP^C^). Prion propagation proceeds in two distinct mechanistic phases: an exponential phase 1, which rapidly reaches a fixed level of infectivity irrespective of PrP^C^ expression level, and a plateau (phase 2), which continues until clinical onset with duration inversely proportional to PrP^C^ expression level. We hypothesized that neurotoxicity relates to distinct neurotoxic species produced following a pathway switch when prion levels saturate. Here we show a linear increase of proteinase K-sensitive PrP isoforms distinct from classical PrP^Sc^ at a rate proportional to PrP^C^ concentration, commencing at the phase transition and rising until clinical onset. The unaltered level of total PrP during phase 1, when prion infectivity increases a million-fold, indicates that prions comprise a small minority of total PrP. This is consistent with PrP^C^ concentration not being rate limiting to exponential prion propagation and neurotoxicity relating to critical concentrations of alternate PrP isoforms whose production is PrP^C^ concentration dependent.

## Abstract (web summary)

Prions (PrP) are infectious agents that cause lethal neurodegenerative diseases. Here the authors study the kinetics of prion propagation in mice and show that the onset of neuropathology occurs during the late phase of disease and is hypothesized to be due to increases in a toxic isoform of PrP that is different from the infectious species.

## Graphical abstract

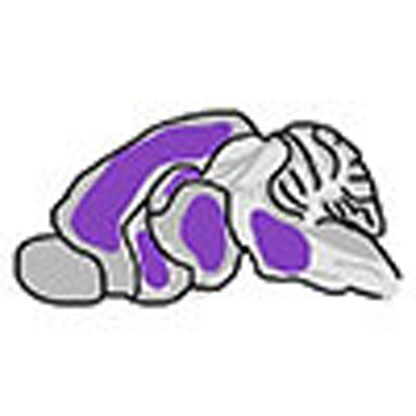

Prions are infectious agents causing lethal neurodegenerative diseases, such as Creutzfeldt–Jakob disease in humans, and scrapie and bovine spongiform encephalopathy in animals, following prolonged asymptomatic incubation periods^1^. In humans, these can span decades and are followed by a rapidly progressive, often stereotypic, clinical phase, which is invariably fatal^1^,^2^. In inbred lines of laboratory mice, incubation periods span months and yet are highly reproducible for a defined line with clinical onsets spanning only a few days.

In studies of mice intracerebrally infected with the Rocky Mountain Laboratory (RML) prion strain, we have previously demonstrated that prion propagation in the brain involves two distinct phases^3^. In phase 1, prions propagate exponentially, not rate limited by cellular prion protein (PrP^C^) concentration, rapidly rising by ~10^6^-fold to reach a maximal prion titre, which is also independent of PrP^C^ concentration over the range we studied. This is followed by a plateau phase (phase 2), which determines time to clinical onset of disease, the duration of which is inversely proportional to PrP^C^ concentration. Determination of prion titres at the beginning and end of the plateau in FVB/N mice by mouse end-point titration bioassay showed indistinguishable levels of infectivity^3^. Prion propagation and neurotoxicity can therefore be uncoupled, arguing that prions are not themselves directly neurotoxic. We hypothesized that once prion propagation saturates, there is a mechanistic switch from autocatalytic production of infectivity (phase 1) to a toxic pathway where prions act as a catalytic surface for the production of toxic species in a process now linearly dependent on PrP^C^ concentration^3^. The rate of production of such toxic PrP species (previously designated PrP^L^ for lethal^4^) would determine the time to exceed local toxic thresholds and subsequent clinical onset.

According to the widely accepted protein-only hypothesis^5^, prions are thought to comprise multi-chain forms of misfolded, host-encoded, PrP^C^, referred to as PrP^Sc^ (refs 6, 7) and are now thought to propagate by an autocatalytic process of seeded fibrilization and fission^8^,^9^,^10^. It is increasingly recognized that such processes may be involved in the pathogenesis of commoner neurodegenerative conditions, including Alzheimer’s and Parkinson’s diseases^11^.

The multi-chain forms of misfolded prion protein thought to comprise infectious prions have been generally referred to as PrP^Sc^ and originally defined by their relative protease-resistance (with characteristic band shift on western blotting due to deletion of an amino-terminal fragment following treatment with proteinase K (PK)) and detergent insolubility as compared with normal PrP^C^ (refs 7, 12). However, it is becoming clear that there are multiple disease-related forms of PrP, some of which are protease sensitive^13^,^14^. Indeed, such protease-sensitive disease-related PrP may constitute the majority of infectivity in some prion isolates^14^. These species remain poorly defined in physical terms and an internationally agreed, even provisional, nomenclature is lacking. As PrP^Sc^ is by definition protease resistant, we do not favour the term protease-sensitive PrP^Sc^ (sPrP^Sc^) (ref. 15). Here we simply use the term ‘PK-sensitive disease-related PrP’ to refer to the ensemble of PrP species seen in prion infection but which do not meet the biochemical definition of ‘classical’ PrP^Sc^.

In our previous studies of the kinetics of prion propagation, we determined the brain prion titre at defined intervals during the incubation period in four types of mice with different PrP^C^ expression levels^3^. We have now proceeded to measure changes in disease-related PrP isoforms in the brain in mice in both the original^3^ and repeat time-course studies. We have developed an assay to measure total PrP. This must comprise PrP^C^ and all disease-related isoforms (which are the sum of PK-sensitive and PK-resistant (classical PrP^Sc^) isoforms). By subtraction of levels of classical PK-resistant PrP^Sc^, any rise would represent the ensemble of PK-sensitive disease-related PrP, assuming PrP^C^ levels are unchanged during infection. *Prnp* expression is known to be unaltered during prion infection^16^,^17^. A recent study reported an apparent fall in PrP^C^ concentration towards the end of the incubation period^18^; if confirmed, this would suggest such a subtraction method would underestimate the rise in levels of PK-sensitive disease-related PrP late in phase 2. We have then sought to determine how changes in levels of disease-related isoforms related to the mechanistic phases we have described and to the development of neuropathology.

## Results

### Levels of total PrP and classical PrP^Sc^ during prion infection

**Figure 1:**
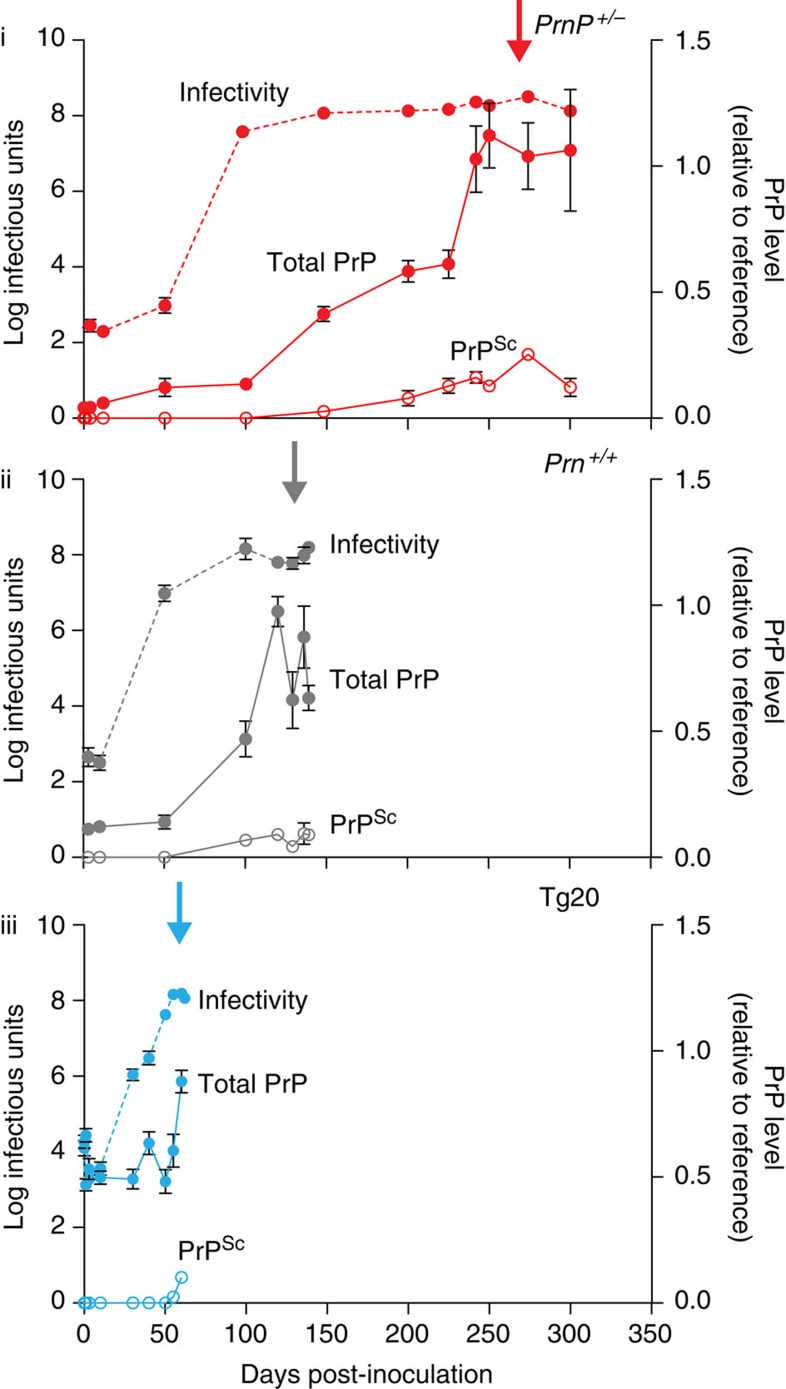
Levels of total PrP and PrP^Sc^ in the brain during prion infection. Three different lines of FVB/N mice: *Prnp*^+/0^ with 50% wild-type PrP^C^ expression (i); *Prnp*^+/+^ with wild-type PrP^C^ expression (ii); and Tg20 with ~eightfold overexpression of PrP^C^ (iii) were intracerebrally infected with RML prions. Mean incubation periods are indicated by arrows and were (in days±s.d.): *Prnp*^+/0^, 258±23.5; *Prnp*^+/+^, 137±1.5; and Tg20, 59.5±2.0. Mice were culled at defined time points or at clinical onset of disease. Prion titres (log tissue-culture infectious units per gram brain; bars indicate s.e.m. and in some cases are smaller than the symbols used to designate mean) are as described previously^3^ (closed circles; dotted lines). Total PrP comprising PrP^C^, classical PK-resistant PrP (PrP^Sc^) and disease-related but PK-sensitive PrP isoforms were determined by ELISA (closed circles; solid lines). In addition, PrP^Sc^ was quantified after PK digestion by ELISA (open circles; solid lines). Total PrP and PrP^Sc^ levels are presented as a ratio relative to a clinical end point reference; bars indicate s.e.m and, in some cases, are smaller than the symbol used to designate mean; group sizes were three to six.

**Figure 2:**
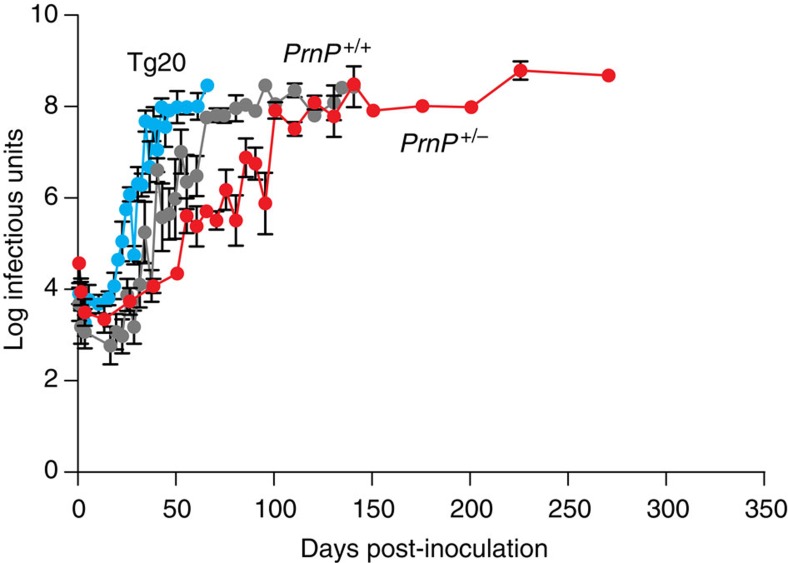
Repeat kinetic time courses showing infectivity in three different lines of mice after prion infection with a high saturation of time points. Three different lines of FVB/N mice: Tg20 with ~eightfold overexpression of PrP^C^ in blue, *Prnp*^+/+^ with wild-type PrP^C^ expression in grey and *Prnp*^+/0^ with 50% wild-type PrP^C^ expression in red, were infected with RML prions. Mice were culled at defined time points or at onset of disease and prion titres determined using scrapie cell assay (SCA) or scrapie cell assay in end-point format (SCEPA) for low-titre samples. Prion titres (log tissue-culture infectious units per gram brain; bars indicate s.e.m. and, in some cases, are smaller than the symbols used to designate mean) are closely similar to those described previously^3^. Normal interval regression was used to calculate mean and s.e.m for timed culls where one or more samples were below assay sensitivity cut-off. This only applied to samples up to day 37 of the time courses. All samples from three timed culls in the *PrnP*^+/+^ time course (5, 10 and 13 days) were below assay sensitivity cut-off and are not shown.

In our previous study^3^, we intracerebrally inoculated large groups of the following mice with RML mouse prions (30 μl 1% brain homogenate containing 10^5.8^ intracerebral LD_50_ units) and groups of five to six mice were killed at multiple defined time points or at the onset of clinical disease: inbred FVB/N mice (*Prnp*^*+/+*^; wild-type PrP^C^ expression level); *Prnp*-null mice^19^ (*Prnp*^o/o^; no PrP^C^ expression; FVB/N background); hemizygous *Prnp*-null mice (*Prnp*^*+/*o^; 50% wild-type PrP^C^ expression; FVB/N background) and Tg20 transgenic mice^20^ (~8-fold wild-type PrP^C^ expression level; FVB/N background). Prion titre in mouse brain was determined by scrapie cell assay (SCA) or SCA in end-point format (scrapie cell endpoint assay (SCEPA)) for low-titre samples as previously reported^21^ (Fig. 1). Prions were undetectable in *Prnp*^o/o^ mice, which are unable to propagate prions^22^, after 10 days, indicating clearance of the inoculum by this time and allowing interpretation of *de novo* produced prions in the other mouse lines^3^. In our earlier work, we had anticipated that prion titres would principally rise late in the incubation period such that relatively few timed culls were early in the incubation period^3^. We have since repeated the original study with many more timed culls, particularly in phase 1. The infectivity curves obtained were closely similar to our earlier results (Fig. 2).

We then proceeded to measure changes in disease-related PrP isoforms in the brain in these mice to determine how these related to the mechanistic phases we have described and to the development of neuropathology. Although our ultimate aim is to isolate and structurally characterize the putative toxic PrP species (PrP^L^), at this stage we do not know whether toxicity is mediated by a single defined species of high specific toxicity, or, at the other extreme, relates to an ensemble of diverse species with a generic toxicity. It appears that infectious prion strains constitute a cloud or quasispecies^10^,^23^,^24^, and it is possible that infectious and toxic PrP populations overlap. Given these uncertainties and the lack of physical characterization of disease-related PrP species, we modified for our purposes an enzyme-linked immunosorbent assay (ELISA) that is capable of detecting all PrP chains following their denaturation with heat and SDS^14^,^25^,^26^. This assay therefore provides a measure of total PrP present in brain homogenates and must necessarily comprise the sum of normal PrP^C^ and disease-related PrP generated during the infection. The latter would comprise both classical PK-resistant PrP^Sc^ and PK-sensitive disease-related PrP isoforms^13^,^14^,^26^. As *Prnp* expression is unaltered^16^,^17^ and PrP^C^ concentration does not rise^18^ during prion infection, the rise in total PrP represents the rise in levels of total disease-related PrP (including PK-resistant and PK-sensitive forms). Levels of classical PK-resistant PrP^Sc^ were also determined, thus enabling estimation of PK-sensitive disease-related PrP isoform levels during the incubation period (Fig. 1).

**Figure 3:**
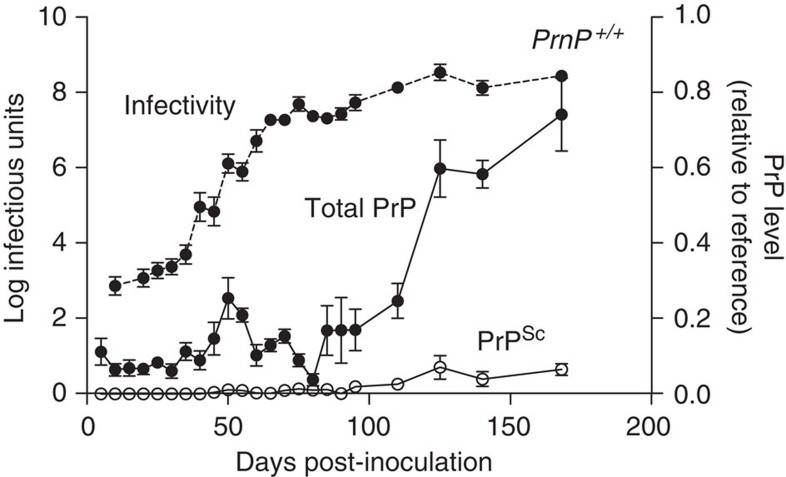
Repeat time course of RML prion infection in FVB/N wild-type mice used for detailed neuropathological analysis. FVB/N mice were infected with RML prions and the mean incubation period for these mice was (in days±s.d.) 168±2.5. Mice were culled at defined time points or at onset of disease and prion titres were determined using scrapie cell assay (SCA) or scrapie cell assay in end-point format (SCEPA) for low-titre samples. Prion titres (log tissue-culture infectious units per gram brain; bars indicate s.e.m. and, in some cases, are smaller than the symbols used to designate mean) are closely similar to those described previously^3^ (closed circles; dotted lines). Normal interval regression was used to calculate mean and s.e.m for timed culls where one or more samples were below assay sensitivity cut-off. This only applied to samples up to day 30 of the time course. All samples from two timed culls in the infectivity time course (5 and 15 days) were below assay sensitivity cut-off and therefore are not shown. Total PrP comprising PrP^C^, classical PK-resistant PrP (PrP^Sc^) and disease-related but PK-sensitive PrP isoforms were determined using ELISA (closed circles; solid lines). In addition, PrP^Sc^ was quantified after PK digestion by ELISA (open circles; solid lines). Total PrP and PrP^Sc^ levels are presented relative to a clinical end-point reference; bars indicate s.e.m and, in some cases, are smaller than symbol used to designate mean; group sizes were four to six.

Remarkably, total PrP levels, although starting at different levels in the three mouse lines due to their different PrP^C^ expression levels, were essentially unchanged throughout phase 1 in wild-type, *Prnp*^*+/*o^ and Tg20 mice, a period during which titres rose in each case by ~10^6^-fold (Fig. 1). When prion titre had reached plateau (marking the start of phase 2), total PrP levels then rose in a linear manner throughout phase 2 with a gradient approximately proportional to PrP^C^ expression level (Fig. 1). In agreement with previous studies, classical PrP^Sc^ represents only a small fraction of total PrP levels at onset of clinical disease and the majority of this is detected towards the end of phase 2 (Figs 1 and 3). Notably, in all three lines of mice, clinical onset appears to ensue only once a closely similar level of total disease-related PrP in the brain is reached (Figs 1 and 3). These data suggest that a particular PrP isoform(s) that is generated in phase 2 must reach a critical threshold level to elicit neurotoxic effects, irrespective of the level of PrP^C^ expressed in the brain.

### Correlation with onset of neuropathological changes

**Figure 4:**
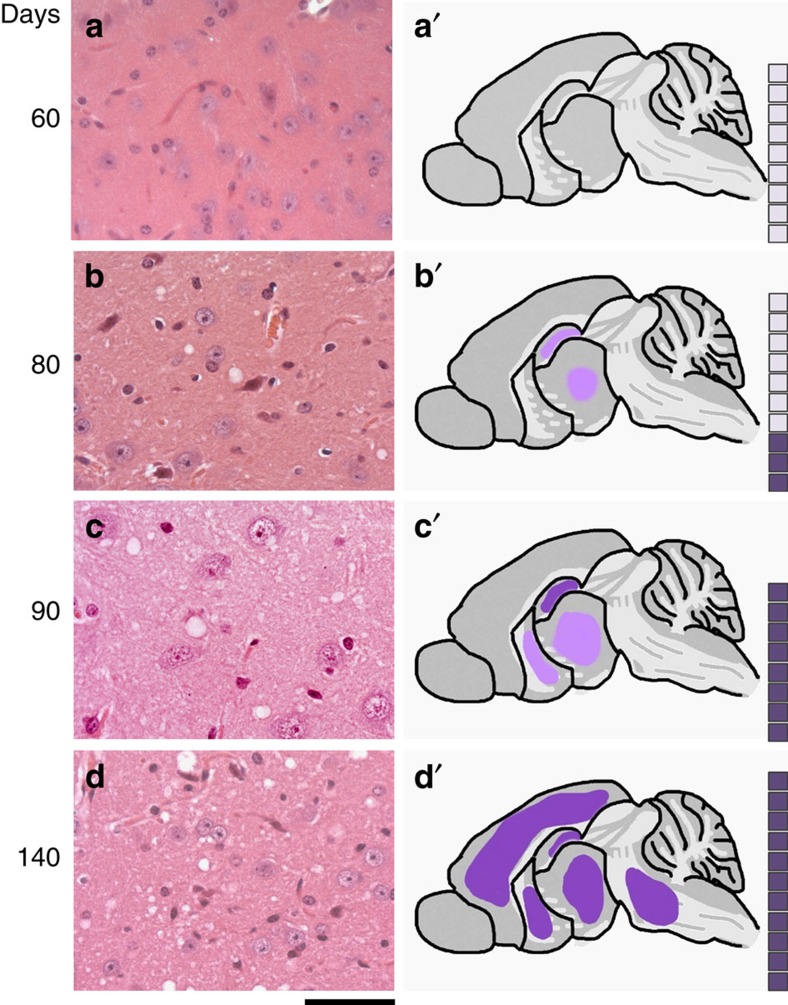
Temporal development of spongiform neuropathology during prion infection. FVB/N mice were intracerebrally infected with RML prions and the mean incubation period was (in days±s.d.) 168±2.5. Mice were culled at defined time points or at onset of clinical disease and brain sections stained with haematoxylin and eosin (H&E). Spongiform vacuolation was absent in all mice at day 60 (**a**), present at a mild level in the thalamus and hippocampus in 3/10 mice at day 80 (**b**) and in 8/8 mice at day 90 (**c**). By day 140, all mice showed moderate and widespread spongiosis affecting the cortex, midbrain and basal ganglia, in addition to hippocampus and thalamus (**d**). (**a**–**d**) H&E staining in the thalamus. (**a**’–**d**’) Schematics showing regional distribution of spongiosis. The boxes correspond to the number of mice in each group and the dark shaded boxes represent the number of animals positive for the neuropathology described. Scale bar, 50 μm (**a**–**d**).

**Figure 5:**
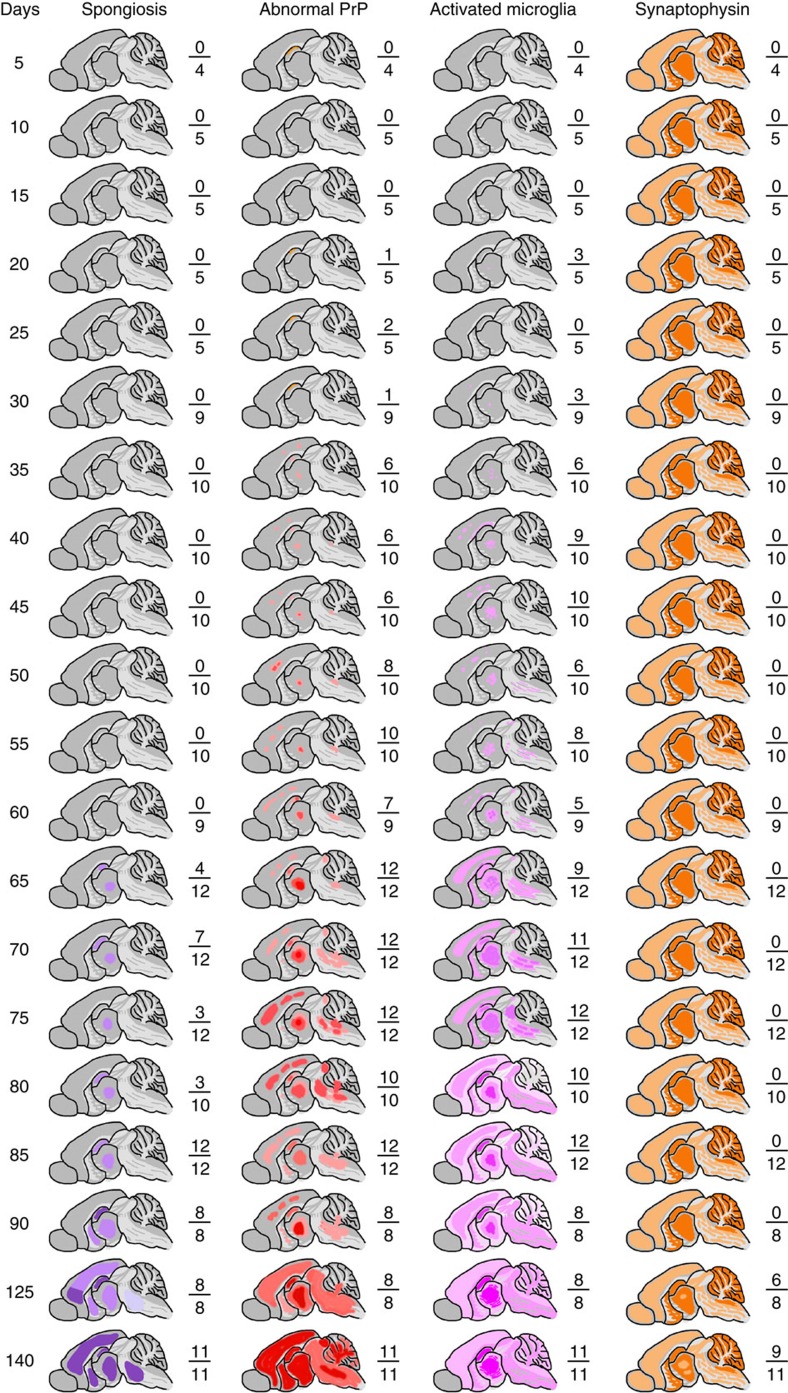
Evolution of pathological changes in the brain during prion infection. FVB/N mice were intracerebrally inoculated with RML prions and groups of mice culled at defined time points or at onset of clinical prion disease. The mean incubation period was 168±2.5 days (±s.d.). Fixed brains from each timed cull were analysed for spongiform change by haematoxylin and eosin (H&E) staining together with immunohistochemical analyses for abnormal PrP deposition, microglia activation and neuronal loss (monitored by synaptophysin staining). Schematic representations of the brain taken at various time points show the evolution of pathological changes throughout the disease course. Numbers shown next to the schematics report the number of animals in the group positive for the pathology shown as a fraction of the total number of animals in each group.

We then sought to correlate changes in prion titre and disease-related PrP isoforms with onset of neuropathological changes. To allow a systematic neuropathological analysis, we repeated the original time course using only the wild-type FVB mice and performed timed culls with a considerably increased number of time points (at 5-day intervals from post-inoculation day 5 to day 90, then at days 125 and 140) to prepare suitable fixed brain tissues for histology and immunohistochemistry. All neuropathological scoring was performed blind to sample identity. Prion titres were also determined again at the same time points to ensure comparability with the original experiment (Fig. 3). Prion titre had reached plateau level by day 75. Spongiform vacuolation of the neuropil is a key specific histological characteristic of prion disease. It appears to correlate with early disease activity and indeed early spongiosis is completely reversible on targeted knockout of PrP^C^ expression in neuroinvasive RML prion infection of FVB mice^27^. Uniform (12/12 mice), albeit mild, spongiosis was only seen at post-infection day 85 and was restricted to the thalamus and hippocampus. As expected for the RML prion strain, spongiosis then extended to the basal ganglia by day 90 and had become of moderate severity and involved virtually all grey matter areas by day 140 (Figs 4 and 5). No spongiform vacuolation was detected in any mice before post-infection day 65 when small subtle vacuoles in the hippocampus and thalamus were seen in 4/12 animals on haematoxylin and eosin-stained sections (Fig. 5). The uniform appearance of specific spongiform pathology only in phase 2 is consistent with a lack of direct neurotoxicity of infectious prions and the hypothesized production of toxic species via a distinct pathway once prion propagation has saturated. PrP immunohistochemistry using anti-PrP monoclonal antibody ICSM35 revealed evidence of residual inoculum in some animals from day 5 to 30, but without synaptic PrP deposition that is characteristic of prion infection. Very subtle occasional synaptic deposits^28^ were seen in some mice from day 35 to 50; consistent deposits were seen in the thalamus and cortex in all mice from day 55 (Fig. 5). Whether these subtle PrP deposits seen in phase 1 simply represent infectious prions is unknown. Microglia were assessed using antibody Iba1 and the earliest reproducible activation was seen at day 35 in the thalamus in some animals. Activation was however well established in all animals by day 65. Synaptophysin immunoreactivity was assessed to detect synaptic loss. The first clear reduction of synaptic density and granular normality was detected at day 125 (Fig. 5).

## Discussion

The basis of neurotoxicity in prion neurodegeneration, its relationship to the prolonged clinically silent incubation periods and the remarkable synchronicity of clinical onset have remained obscure. Although classical PrP^Sc^ rises towards the end of the incubation period and has been considered a candidate neurotoxic species, the well-documented existence of subclinical carrier states of prion infection, where conventional mice with normal PrP^C^ expression live a typical lifespan despite harbouring levels of classical PrP^Sc^ and prion titres similar to those of mice with end-stage clinical disease^4^,^29^,^30^,^31^,^32^,^33^, argues against this.

**Figure 6:**
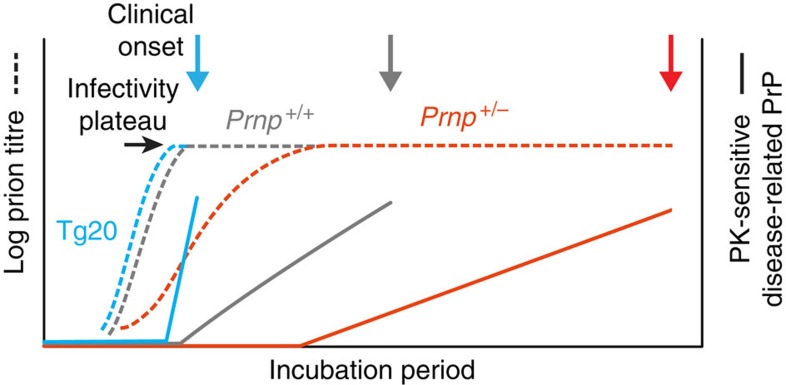
Diagrammatic representation of kinetics of prion propagation and toxicity. Prion replication (dotted lines) is exponential in phase 1 until a limiting titre of prions (infectivity plateau) is attained independent of PrP^C^ expression level. Plateau length (phase 2) is inversely proportional to PrP^C^ expression level^3^. During phase 1, total PrP levels do not significantly change from the starting PrP^C^ level before infection (Fig. 1). PK-sensitive disease-related PrP is total PrP minus starting PrP^C^ level (by definition zero in all three mouse lines before infection) and classical PrP^Sc^. At the phase transition, PK-sensitive disease-related PrP isoforms begin to accumulate (solid lines). The rise of PK-sensitive disease-related PrP appears linear and approximately proportional to mouse PrP^C^ expression level (Figs 1 and 3). Onset of clinical disease occurs when a critical level of toxic species is reached, which is closely similar in each mouse line.

These findings are consistent with the general model of prion propagation and neurotoxicity in which prion infectivity and toxicity are mediated by different PrP species, which are the product of distinct pathways^3^,^10^. According to this model, production of neurotoxic species is triggered when the exponential phase (phase 1) of prion propagation saturates (following depletion of a key co-factor or occupancy of available replication sites). The fact that this explosive rise in prion titre is not rate limited by PrP^C^ concentration is consistent with the absence of a rise of total PrP during phase 1 arguing that infectious prions comprise a small minority of available PrP. In phase 2, there is a linear rise in PK-sensitive disease-related PrP isoforms, a mechanistically distinct process rate limited by, and directly proportional to, PrP^C^ concentration. Clinical onset occurs at a similar level of PK-sensitive disease-related PrP isoforms irrespective of PrP^C^ expression level (Fig. 6). The classical neuropathological features of prion disease, indicative of the presence of neurotoxic species, are only detected once phase 2 is well established. The precise temporal relationship between production of such neurotoxic species, reaching critical concentrations at critical sites, and onset of neuropathology cannot be defined by these experiments, and will ultimately require isolation of PrP^L^ species and direct studies of neurotoxicity. However, such isolation and physical characterization of neurotoxic species, as with other neurodegenerative diseases, is likely to prove technically demanding. Such species may be transitory and inherently labile. In addition, as discussed above, it is unknown whether the majority of the PK-sensitive disease-related PrP isoforms constitute generic toxic material or whether at the other extreme a low abundance specific sub-species of defined structure and receptor interaction with high specific toxicity constitutes the hypothetical PrP^L^. An essential prerequisite will be the development of a robust *in vitro* assay for such neurotoxic activity with a sufficient dynamic range to facilitate fractionation and isolation of such species. These data also suggest that the term PrP^Sc^, often used synonymously with infectivity, should be restricted to material as classically biochemically defined^7^,^12^. Such classical PrP^Sc^ only constitutes a small (variable) fraction of total disease-related PrP isoforms and its proportional contribution to both infectivity and neurotoxicity remains unclear.

All the common neurodegenerative diseases involve accumulation of aggregated misfolded host proteins and it is increasingly proposed that seeded protein aggregation (or ‘prion-like mechanisms’) may be involved in their pathogenesis and/or spread of pathology throughout the neuraxis. Experimental prion disease offers major advantages in studying neurodegenerative processes, because prion diseases naturally affect many mammalian species, including laboratory rodents, avoiding the inherent limitations of mouse models of other neurodegenerative diseases and, crucially, because of the predictable incubation periods and tools established to determine prion strain properties and accurately measure titre^1^. If comparable tools can be developed for the commoner neurodegenerative conditions such as Alzheimer’s and Parkinson’s diseases, it will be interesting to investigate whether similar mechanistic relationships between seeding activity and neurotoxicity are also involved.

## Methods

All experimental procedures involving prions were carried out in microbiological containment level 3 facilities with strict adherence to safety protocols.

### Prion inoculation of mice

Work with mice was performed under licence granted by the UK Home Office and conformed to the University College London institutional and ARRIVE guidelines. RML prion inoculum (I6200) was prepared and titrated^14^, and mice (female 6–9 weeks old) were intracerebrally inoculated and monitored as described previously^3^. Groups of 5–12 mice were killed at multiple defined time points or at onset of clinical disease. Brains were removed and divided sagittally with half-frozen at −70 °C and half-fixed in 10% (v/v) formal buffered saline. Brain homogenates (10% (w/v)) were prepared from frozen specimens in sterile Dulbecco’s PBS lacking Ca^2+^ and Mg^2+^ ions (D-PBS) and stored as aliquots at −70 °C.

### Prion bioassay

PK1 cells (sub-clone PK1/2), a line derived from N2a cells that are highly susceptible to RML prions^21^ were routinely grown into OFCS medium (Opti-MEM, containing 10% FCS; 100 U ml^−1^ penicillin and 100 μg ml^−1^ streptomycin; Invitrogen, UK) using 15 cm Petri dishes. Prion infectivity was assayed using two modified automated protocols based on the original published methods^21^, the SCEPA and the automated SCA (ASCA). The SCEPA is capable of detecting sample infectivity in a range down to ~10^3^ tissue culture infectious units (TCIU)  ml^−1^ of 10% (w/v) brain homogenate^21^, while the ASCA is typically used to detect infectivity above 10^4.5^ TCIU ml^−1^ in 10% (w/v) brain homogenate. The day before infection, PK1/2 cells were seeded into 96-well plates (18,000 cells in 260 μl OFCS; Costar flat bottom 96-well plates; Corning, UK) and kept at 37 °C in a 5% CO_2_ incubator. The following day, 10 μl of brain homogenate appropriately diluted in OFCS was applied to the cells. A serial dilution of a reference 10% (w/v) RML brain homogenate (maximal titre of 10^7.7^ TCIU ml^−1^ determined by SCEPA) was applied in parallel. Typically, 10% (w/v) brain homogenate was used in the range of 1 × 10^−3^ to 1 × 10^−5^ dilution for SCEPA and 3 × 10^−4^ to 3 × 10^−6^ dilution for ASCA. Three days after infection, an automated platform (Biomek FX liquid handling robot; Beckman Coulter) was used to split cells. For the SCEPA protocol, cells were split into 96-well plates containing fresh OFCS at ratios of 1:3 on days 3, 5 and 7, followed by splitting 1:8 at days 10, 13, 17 and 21 post infection, while the ASCA was split at a ratio of 1:8 on days 3, 6, 10 and 14 post infection. ELISpot plates (96-well– polyvinylidene difluoride membrane plates; Millipore, UK) were prepared from the SCEPA assays on days 17 and 21 post infection and on days 10 and 14 post infection for the ASCA assays (25,000 cells per well). The plates were vacuum drained and dried at 50 °C after which 60 μl of 1 μg ml^−1^ PK (Roche, UK) in lysis buffer (50 mM Tris HCl, pH 8, containing 150 mM NaCl, 0.5% (w/v) sodium deoxycholate and 0.5% (v/v) Triton X-100) was added to each well and incubated for 60 min at 40 °C. The plates were washed (2 × 160 μl PBS) after which 120 μl of 3 M guanidinium thiocyanate prepared in 10 mM Tris HCl, pH 8.0, was added to each well for 20 min. The wells were washed (7 × 160 μl PBS) and 150 μl of Superblock dry blend blocking buffer (Perbio, UK) was added to each well and incubated for 1 h. Following vacuum removal of Superblock, each well was incubated with 0.55 μg ml^−1^ anti-PrP monoclonal antibody ICSM18 (D-Gen Ltd, London) prepared in TBST (10 mM Tris HCl, pH 8, 150 mM NaCl, 0.1% (v/v) Tween 20) containing 1% (w/v) non-fat dry milk for 1 h. After washing (5 × 160 μl TBST), wells were incubated for 1 h with 60 μl goat anti-mouse alkaline phosphatase-conjugated anti-IgG1 (Southern Biotechnology Associates, USA) diluted 1:8,000 in TBST-1% (w/v) non-fat dry milk. Following washing (5 × 160 μl TBST), wells were incubated for 35 min with 50 μl AP dye (Bio-Rad, USA). The plates were then washed twice with water, dried and stored at −20 °C. Spot counts (reporting PK-resistant PrP-positive cells) were determined with a Zeiss KS Elispot system (Stemi 2000-C stereo microscope equipped with a Hitachi HV-C20A colour camera, a KL 1,500 LCD scanner and Wellscan software from Imaging Associates, Oxfordshire, UK). Normal interval regression was used to calculate mean and s.e.m. for the groups that contained points below assay sensitivity cut-off. If all samples in a timed group were below assay sensitivity cut-off, the group was excluded from further analyses.

### ELISA for total PrP

PrP levels in brain homogenate were determined by ELISA as previously described^14^,^26^, but with adaptations. A pooled preparation of 10% (w/v) terminal RML mouse brain homogenate (I8700) of known prion infectivity titre^26^ was diluted into D-PBS. These samples were used to generate standard curves of fluorescence versus I8700 concentration, and were processed in parallel with unknown samples. PrP levels in unknown samples were expressed relative to the I8700 reference preparation. The protein concentration of 10% (w/v) brain homogenate was determined using the Bicinchoninic acid assay kit (Thermo Scientific, Rockford, IL, USA) according to the manufacturer’s instructions and aliquots subsequently adjusted with D-PBS to give a final protein concentration of 7 mg ml^−1^. Twenty-microlitre aliquots of these samples were treated with an equal volume of either D-PBS alone or D-PBS containing 50 μg ml^−1^ PK and incubated at 37 °C for 1 h. This concentration of PK (at a ratio of 1:140 total brain protein by weight) was used to ensure complete digestion of PrP^C^ in overexpressing Tg20 transgenic mice and to retain only the most PK-resistant abnormal PrP conformers classically designated as PrP^Sc^^7^. After addition of 1 μl 100 mM 4-(2-aminoethyl)-benzenesulphonyl fluoride prepared in water and 1 μl 20% (w/v) SDS prepared in water, the samples were heated at 100 °C for 10 min. Twenty microlitres of the denatured samples were then diluted into ELISA capture buffer (50 mM Tris/HCl, pH 8.4, containing 2% (v/v) Triton X-100, 2% (w/v) sodium lauroylsarcosine and 2% (w/v) BSA (fraction V, protease free, Sigma)) between 30 and 150 times for analysis. The use of multiple dilutions ensured that fluorescence measurements generated by unknown samples could be correlated with the linear range of the standard curves of I8700. Microtitre plates (Microlon 96W, Greiner Bio-One) were coated overnight with anti-PrP monoclonal antibody ICSM18 (D-Gen Ltd) diluted in carbonate coating buffer (250 ng antibody per well). The wells were blocked using Superblock T20 (PBS) blocking buffer (Thermo Scientific) and incubated with constant agitation for 1 h at 37 °C. Aliquots (50 μl) of the diluted samples were transferred into the wells of the plates and incubated with constant agitation at 37 °C for 1 h. Wells were washed with 3 × 300 μl of PBS containing 0.05% (v/v) Tween 20 using an automated microplate washer, followed by the addition of 100 μl of PBS containing 1% (v/v) Tween 20 and 1 μg ml^−1^ biotinylated anti-PrP monoclonal antibody ICSM 35 (D-Gen Ltd). Following incubation at 37 °C for 15 min with constant agitation, wells were washed as detailed above, followed by the addition of 100 μl of PBS containing 1% (v/v) Tween 20 and a dilution of streptavidin–horseradish-peroxidase conjugate (1:10,000 dilution; Thermo Scientific). After incubation at 37 °C for 15 min with constant agitation, wells were washed with 4 × 300 μl of PBS containing 0.05% (v/v) Tween 20. Wells were developed using QuantaBlu Fluorogenic Peroxidase substrate kit according to the manufacturer’s instructions (Thermo Scientific). Fluorescence was measured on a Tecan infinite M200 spectra image microplate reader (λex=313 nm, λem=398 nm).

### Neuropathology and immunohistochemistry

Brain fixed in 10% buffered formol-saline was immersed in 98% formic acid for 1 hour and paraffin wax embedded. Serial sections (4-μm-thick) were pretreated by boiling for 10 min in a low ionic strength buffer (2.1 mM Tris, 1.3 mM EDTA, 1.1 mM sodium citrate, pH 7.8) before exposure to 98% formic acid for 5 min. Abnormal PrP accumulation was examined using anti-PrP monoclonal antibody ICSM 35 (D-Gen Ltd). Microglial activation was examined using an ionized calcium binding adaptor molecule 1 (Iba1) antibody (Wako Pure Chemical Industries, Ltd) and synaptophysin was examined using rabbit polyclonal antibodies (Z66, Life Technologies). All immunohistochemistry was performed on automated immunohistochemistry staining machines (Ventana Medical Systems Inc., Tucson, Arizona) using proprietary secondary detection reagents (Ventana Medical Systems Inc.) before development with 3′3-diaminobenzedine tetrachloride as the chromogen^34^. Conventional methods were used for Harris haematoxylin and eosin staining. Appropriate positive and negative controls were used throughout. Photographs were taken on an ImageView digital camera and composed with Adobe Photoshop.

## Author contributions

M.K.S., H.A.-D., B.S., M.W.D.O., A.R.-L., S.L. and C.S. performed the work. J.M.L. and S.B analysed the neuropathology. M.K.S., A.R.C., J.D.F.W. and J.C. designed the study and analysed the data. J.C. drafted the manuscript. All authors discussed the results and commented on the manuscript.

## Additional information

**How to cite this article:** Sandberg, M. K. *et al.* Prion neuropathology follows the accumulation of alternate prion protein isoforms after infective titre has peaked. *Nat. Commun.* 5:4347 doi: 10.1038/ncomms5347 (2014).

## References

[b1] Collinge J. Prion diseases of humans and animals: their causes and molecular basis. Annu. Rev. Neurosci. 24, 519–550 (2001). 11283320 10.1146/annurev.neuro.24.1.519

[b2] Collinge J. *et al.* Kuru in the 21st century--an acquired human prion disease with very long incubation periods. Lancet 367, 2068–2074 (2006). 16798390 10.1016/S0140-6736(06)68930-7

[b3] Sandberg M. K., Al Doujaily H., Sharps B., Clarke A. R. & Collinge J. Prion propagation and toxicity *in vivo* occur in two distinct mechanistic phases. Nature 470, 540–542 (2011). 21350487 10.1038/nature09768

[b4] Hill A. F. *et al.* Species barrier independent prion replication in apparently resistant species. Proc. Natl Acad. Sci. USA 97, 10248–10253 (2000). 10963685 10.1073/pnas.97.18.10248 PMC27848

[b5] Griffith J. S. Self replication and scrapie. Nature 215, 1043–1044 (1967). 4964084 10.1038/2151043a0

[b6] Prusiner S. B. Novel proteinaceous infectious particles cause scrapie. Science 216, 136–144 (1982). 6801762 10.1126/science.6801762

[b7] Prusiner S. B. Prions. Proc. Natl Acad. Sci. USA 95, 13363–13383 (1998). 9811807 10.1073/pnas.95.23.13363 PMC33918

[b8] Gajdusek D. C. Transmissible and non-transmissible amyloidoses: autocatalytic post-translational conversion of host precursor proteins to beta- pleated sheet configurations. J. Neuroimmunol. 20, 95–110 (1988). 3143742 10.1016/0165-5728(88)90140-3

[b9] Come J. H., Fraser P. E. & Lansbury P. T. J. A kinetic model for amyloid formation in the prion diseases: importance of seeding. Proc. Natl Acad. Sci. USA 90, 5959–5963 (1993). 8327467 10.1073/pnas.90.13.5959 PMC46846

[b10] Collinge J. & Clarke A. A general model of prion strains and their pathogenicity. Science 318, 930–936 (2007). 17991853 10.1126/science.1138718

[b11] Jucker M. & Walker L. C. Self-propagation of pathogenic protein aggregates in neurodegenerative diseases. Nature 501, 45–51 (2013). 24005412 10.1038/nature12481 PMC3963807

[b12] Meyer R. K. *et al.* Separation and properties of cellular and scrapie prion proteins. Proc. Natl Acad. Sci. USA 83, 2310–2314 (1986). 3085093 10.1073/pnas.83.8.2310 PMC323286

[b13] Safar J. *et al.* Eight prion strains have PrP^Sc^ molecules with different conformations. Nat. Med. 4, 1157–1165 (1998). 9771749 10.1038/2654

[b14] Cronier S. *et al.* Detection and characterization of proteinase K-sensitive disease-related prion protein with thermolysin. Biochem. J. 416, 297–305 (2008). 18684106 10.1042/BJ20081235 PMC2584334

[b15] Tremblay P. *et al.* Mutant PrP(Sc) conformers induced by a synthetic peptide and several prion strains. J. Virol. 78, 2088–2099 (2004). 14747574 10.1128/JVI.78.4.2088-2099.2004 PMC369494

[b16] Hwang D. *et al.* A systems approach to prion disease. Mol. Syst. Biol. 5, 252 (2009). 19308092 10.1038/msb.2009.10 PMC2671916

[b17] Zampieri M., Legname G., Segre D. & Altafini C. A system-level approach for deciphering the transcriptional response to prion infection. Bioinformatics 27, 3407–3414 (2011). 22016408 10.1093/bioinformatics/btr580

[b18] Mays C. E. *et al.* Prion disease tempo determined by host-dependent substrate reduction. J. Clin. Invest. 124, 847–858 (2014). 24430187 10.1172/JCI72241 PMC3904628

[b19] Bueler H. *et al.* Normal development and behaviour of mice lacking the neuronal cell-surface PrP protein. Nature 356, 577–582 (1992). 1373228 10.1038/356577a0

[b20] Fischer M. *et al.* Prion protein (PrP) with amino-proximal deletions restoring susceptibility of PrP knockout mice to scrapie. EMBO J. 15, 1255–1264 (1996). 8635458 PMC450028

[b21] Klohn P., Stoltze L., Flechsig E., Enari M. & Weissmann C. A quantitative, highly sensitive cell-based infectivity assay for mouse scrapie prions. Proc. Natl Acad. Sci. USA 100, 11666–11671 (2003). 14504404 10.1073/pnas.1834432100 PMC208815

[b22] Bueler H. *et al.* Mice devoid of PrP are resistant to scrapie. Cell 73, 1339–1347 (1993). 8100741 10.1016/0092-8674(93)90360-3

[b23] Li J., Browning S., Mahal S. P., Oelschlegel A. M. & Weissmann C. Darwinian evolution of prions in cell culture. Proc. Natl Acad. Sci. USA 327, 869–872 (2010). 10.1126/science.1183218 PMC2848070 20044542

[b24] Collinge J. Prion strain mutation and selection. Science 328, 1111–1112 (2010). 20508117 10.1126/science.1190815

[b25] Wadsworth J. D. *et al.* Phenotypic heterogeneity in inherited prion disease (P102L) is associated with differential propagation of protease-resistant wild-type and mutant prion protein. Brain 129, 1557–1569 (2006). 16597650 10.1093/brain/awl076

[b26] D'Castro L. *et al.* Isolation of proteinase k-sensitive prions using pronase E and phosphotungstic acid. PLoS ONE 5, e15679 (2010). 21187933 10.1371/journal.pone.0015679 PMC3004958

[b27] Mallucci G. *et al.* Depleting neuronal PrP in prion infection prevents disease and reverses spongiosis. Science 302, 871–874 (2003). 14593181 10.1126/science.1090187

[b28] Ellison D. *et al.* inNeuropathology. A Reference Text of CNS Pathology 659–693Elseivier Mosby (2013).

[b29] Hill A. F. & Collinge J. Subclinical prion infection. Trends Microbiol. 11, 578–584 (2003). 14659690 10.1016/j.tim.2003.10.007

[b30] Race R., Raines A., Raymond G. J., Caughey B. & Chesebro B. Long-term subclinical carrier state precedes scrapie replication and adaptation in a resistant species: Analogies to bovine spongiform encephalopathy and variant Creutzfeldt-Jakob disease in humans. J. Virol. 75, 10106–10112 (2001). 11581378 10.1128/JVI.75.21.10106-10112.2001 PMC114584

[b31] Thackray A. M., Klein M. A., Aguzzi A. & Bujdoso R. Chronic subclinical prion disease induced by low-dose inoculum. J. Virol. 76, 2510–2517 (2002). 11836429 10.1128/jvi.76.5.2510-2517.2002 PMC153817

[b32] Thackray A. M., Klein M. A. & Bujdoso R. Subclinical prion disease induced by oral inoculation. J. Virol. 77, 7991–7998 (2003). 12829838 10.1128/JVI.77.14.7991-7998.2003 PMC161915

[b33] Asante E. A. *et al.* BSE prions propagate as either variant CJD-like or sporadic CJD-like prion strains in transgenic mice expressing human prion protein. EMBO J. 21, 6358–6366 (2002). 12456643 10.1093/emboj/cdf653 PMC136957

[b34] Wadsworth J. D. *et al.* Molecular diagnosis of human prion disease. Methods Mol. Biol. 459, 197–227 (2008). 18576157 10.1007/978-1-59745-234-2_14

